# The Role of Dopamine in Repurposing Drugs for Oncology

**DOI:** 10.3390/biomedicines11071917

**Published:** 2023-07-06

**Authors:** Catarina Moura, Nuno Vale

**Affiliations:** 1 OncoPharma Research Group, Center for Health Technology and Services Research (CINTESIS), Rua Doutor Plácido da Costa, 4200-450 Porto, Portugal; cafsm13@gmail.com; 2 CINTESIS@RISE, Faculty of Medicine, University of Porto, Alameda Professor Hernâni Monteiro, 4200-319 Porto, Portugal; 3 ICBAS—School of Medicine and Biomedical Sciences, University of Porto, Rua Jorge Viterbo Ferreira, 228, 4050-313 Porto, Portugal; 4 Department of Community Medicine, Information and Health Decision Sciences (MEDCIDS), Faculty of Medicine, University of Porto, Rua Doutor Plácido da Costa, 4200-450 Porto, Portugal

**Keywords:** dopamine, repurposing drugs, oncology, dopamine signaling, dopamine receptors

## Abstract

Dopamine is a neurotransmitter that plays an important role within the brain by regulating a wide variety of cognitive and emotional processes. In cancer, its role is distinct and uncertain, but it is characterized by the interaction with its receptors that may be in the tumor cells; we have examples of different types of cancer with this characteristic, of which breast and colon cancer stand out. It is believed that dopamine and some of its receptors also influence other cellular processes such as cell proliferation, survival, migration, and invasion. The potential of these receptors has allowed the exploration of existing drugs, originally developed for non-oncological purposes, for the possible treatment of cancer. However, regarding the repurposing of drugs for cancer treatment, the role of dopamine is not so straightforward and needs to be clarified. For this reason, this review intends to present concepts associated with twelve drugs reused for oncology based on dopamine and its receptors. Some of them can behave as antagonists and inhibit tumor cell growth leading to cell death. Attention to this group of drugs may enhance the study of other pharmacological conditions such as signaling pathways related to cell proliferation and migration. Modulation of these pathways using drugs originally developed for other conditions may offer potential therapeutic opportunities in oncology. It is important to note that while the repurposing of oncology drugs based on dopamine signaling is promising, further studies are still needed to fully understand the mechanisms involved and determine the clinical efficacy and safety of these approaches.

## 1. Introduction

Dopamine (DA) is one of the major catecholamine neurotransmitters in the central nervous system (CNS), and its levels present in the basal ganglia are well regulated via several mechanisms involving both astrocytes and neurons [1,2]. DA acts via multiple brain pathways, with DA and DA signaling anomalies in these pathways leading to a variety of neurodegenerative, psychiatric, and immunological diseases [3]. While DA hyperactivity results in a disinhibition of specific actions, DA hypoactivity causes a broad inhibition of motor behaviors. These alterations will manifest in a variety of motor control diseases, including Tourette’s syndrome, Parkinson’s disease, ALS, and developmental stuttering. Therefore, this neurotransmitter is involved in several brain activities, including those that control movement, emotion, cognition, reward, and others [2,4].

Dopamine must attach to a specific set of receptors on the plasma membrane of target cells in order to perform its functions. Dopamine receptors (DRs) are members of the GPCR superfamily and are found in high concentrations in the brain, retina, gastrointestinal system, kidney, adrenal glands, heart, sympathetic ganglia, and blood vessels [4,5]. So far, five distinct subtypes of dopamine receptors have been identified: D1, D2, D3, D4, and D5. All dopamine receptors are metabotropic, meaning they produce second messengers that either activate or inhibit certain cell signaling pathways [4].

Antipsychotic medications are commonly used to treat a variety of psychotic diseases. There are two types of antipsychotics: first generation antipsychotics—which were created in the 1950s and are dopamine receptor antagonists, often known as typical antipsychotics—and second generation antipsychotics, which function on other receptors, such as antagonizing serotonin 2A (5-HT2AR) receptors [6]. While first-generation drugs decrease dopaminergic neurotransmission, second-generation drugs block dopamine D2 receptors while also antagonizing serotonin receptors [6].

Several studies have found that patients with schizophrenia who are treated with antipsychotics had a lower cancer incidence than the general population for certain cancer types [6,7]. The discovery that cancer patients who took antipsychotic medicines alongside their antineoplastic treatment had better clinical outcomes first reinforced the hypothesis that DRs could play important roles in tumor growth [8]. Patients receiving antipsychotics had a lower risk of developing gastrointestinal and brain malignancies. This fact demonstrated that antipsychotics may have an anticancer effect [6].

As more studies show that DA signaling in peripheral tissues is disrupted in cancer, this emerging field offers new insights into cancer cell vulnerabilities and highlights the potential of utilizing and repurposing the libraries of dopaminergic ligands and drugs that have emerged from the discovery and development of neuropharmacological drugs. As a result of the time-consuming, costly, and frequently futile nature of medication research, repurposing dopaminergic drugs for cancer therapy has the potential to help both patients and drug developers [9].

In this review, we will highlight the roles of dopamine signaling in physiological processes, as well as explain how the signaling of these neurotransmitters works. We will also investigate the contradictory effects of dopamine in the context of cancer, as wells as the research of several drugs repurposed from dopamine for the use in cancer treatment.

## 2. Synthesis Cascade via Tyr (Tyrosine)

**Figure 1:**
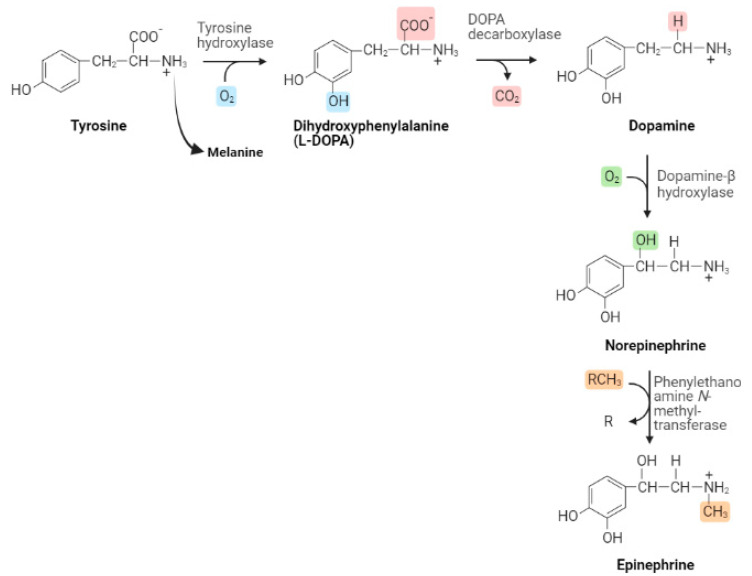
Pathways for biosynthesis of dopamine, a catecholamine neurotransmitter. Image adapted from Purves et al. (2018) [13], using the BioRender on 26 April 2023.

Dopamine, also known as 3,4-dihydroxytyramine, is a neurotransmitter produced by dopaminergic neurons localized in the brain [10]. DA is synthesized by many steps, with the enzyme tyrosine hydroxylase catalyzing the rate-limiting step in this process by converting tyrosine to levodopa (L-DOPA) by adding a hydroxyl group to it (Figure 1). L-DOPA is in turn decarboxylated by amino acid decarboxylase to generate an early form of dopamine which will subsequently be transported by the synaptic vesicle protein VMAT2 (vesicular monoamine transporter 2) into the vesicles. This pre-dopamine transmitter is encapsulated in a vesicle along with other enzymes to continue dopamine synthesis within the transmission vesicles in the presynaptic neurons. After the neuronal activation, DA is released into the synaptic cleft where it will be able to bind to different receptors that are found on the post-synaptic cell membranes. The excess DA is either subsequently recycled back to the presynaptic neuron via dopamine transporters (DAT) or is broken down by monoamine oxidase (MAO) and/or catechol-O-methyltransferase (COMT). This synthesis–release–reabsorption process controls and regulates the physiological dopaminergic responses in response to body demands [2,10]. 

When found inside of the vesicles (captured by the vesicles), dopamine can also be converted into norepinephrine by the enzyme dopamine b-hydroxylase (Figure 1). And, in the adrenal medulla and in some brain regions, norepinephrine can be converted to epinephrine by the enzyme phenylethanolamine N-methyltransferase [11]. They are present in serum at low quantities and can significantly rise in response to acute stress or physical activity. Epinephrine mounts a quick and brief stress response signal in comparison to the more persistent glucocorticoids. Although both norepinephrine and epinephrine bind to adrenergic receptors, their potencies for activation differ. While epinephrine is a powerful activator of β-adrenergic receptor (β-AR), norepinephrine preferentially stimulates the α-AR [12].

**Figure 2:**
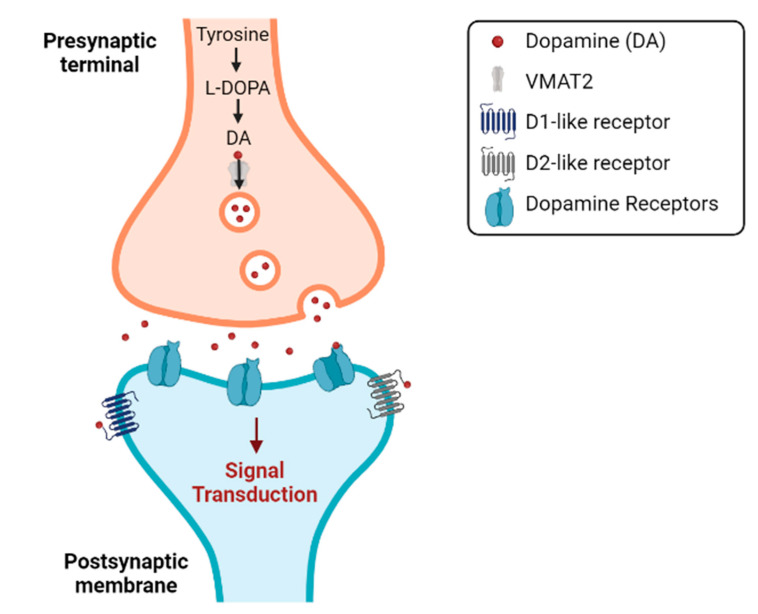
Synthesis of dopamine and its release into the synaptic cleft, which will allow the binding of dopamine to its different receptors, the DRs. Image adapted from Purves et al. (2018) [13], using the BioRender on 14 May 2023.

When DA is transported to and incorporated into the vesicles, it is subsequently released into the synaptic cleft and can then bind to the dopamine receptors (DRs) (Figure 2). In this way, DA can bind to five receptor subtypes: D1–D5, each belonging to members of the G protein-coupled receptor (GPCR) family, which are divided into two large subclasses: D1R-like and D2R-like receptor families [10].

The D1R-like, including D1R and D5R, are receptors that usually bind to proteins that stimulate the enzyme adenylate cyclase (AC), which is responsible for catalyzing the hydrolysis of adenosine triphosphate (ATP) into cyclic adenosine monophosphate (cAMP), consequently increasing the production of cAMP. The cAMP will activate the protein kinase A (PKA), which in turn will phosphorylate the cAMP response element-binding protein (CREB). CREB is subsequently translocated to the nucleus to activate CREB-binding protein (CBP) which will promote the transcription of genes involved in synaptic plasticity. Accordingly, the D1Rs modulate different ion channels, including Ca^2+^, K^+^, and Na^+^ channels and a G protein gated inwardly rectifying K^+^ (GIRK) channel [10].

The D2R, D3R, and D4R are examples of D2R-like receptors, which bind through coupling to proteins that result in the suppression of the AC and PKA-dependent activation pathways as well as the activation of GIRK and the closure of voltage-activated Ca^2+^ channels. By combining with other subtypes, DA receptors can behave as monomers, dimeric, and/or oligomeric complexes. Dimeric and oligomeric complexes have pharmacological and functional traits that are different from those of the individual receptors [10].

In addition, several pathways from the G protein can be activated by DA receptors. The final of these methods is subsequently controlled by multifunctional adaptors of the arrestin protein, which recruits numerous proteins by binding to DA receptors that have been phosphorylated by GPCR kinases (GRKs), including Akt, GSK-3, MAPK, c-Src, Mdm2, and N-ethylmaleimide-sensitive factor. Arrestin’s association with phosphorylated energy receptors inhibits G protein activation as well as promotes receptor endocytosis [10].

As a result, depending on the receptors on the target neuron’s membrane surface and how these neurons react to a rise or decrease in cAMP concentration, dopamine can be both excitatory and inhibitory. Because of this, it is reasonable to anticipate that distinct functional roles for DA will depend on the receptor subtype, cell type, synaptic characteristics, and interactions with other transmitters [10]. 

## 3. Contradictory Effect of Dopamine in Oncology

The discovery of cancer patients taking antipsychotic medications alongside their antineoplastic therapy and the observation that these patients had better clinical outcomes first supported the theory that dopamine receptors may play a key role in the progression of a tumor [8].

According to additional research, patients with Parkinson’s disease or schizophrenia who take ziprasidone, asenapine, quetiapine, clozapine, or aripiprazole (agonists or nonselective antagonists of DRs) or levodopa (a prodrug that results in a non-selective agonism of DRs) have a lower risk of developing a variety of cancers than people in the general population. Nevertheless, the risk of breast cancer is increased in female schizophrenia patients taking DRD2 antagonists with varying selectivity, such as haloperidol, risperidone, paliperidone, or amisulpride [8].

Parkinson’s disease (PD) and cancer have an inverse connection that varies depending on the cancer, according to epidemiological investigations. In a study, a correlation between PD and lung cancer risk was seen, with PD patients alone having a lower probability of a subsequent lung cancer diagnosis. The researchers concluded that this link was nothing more than a bias despite the suggestion that it might be explained by the exposure of PD patients to dopamine medication. Contrarily, other research revealed that melanoma co-occurred with Parkinson’s disease, i.e., that the relative risk for melanoma in patients with PD was seven times higher than expected. The relationship between PD and melanoma, in contrast to lung cancer, is independent of the time of diagnosis; as a result, patients with melanoma have a 50% higher risk of receiving a diagnosis of PD later, and patients with PD are twice as likely to receive a melanoma diagnosis later [9].

There is increasing molecular and clinical evidence indicating a direct biological association between peripheral DA signaling and cancer in terms of risk, development, and progression, despite the fact that epidemiological studies show that the relationship between DA signaling and cancer is complex and contradictory [9].

DRs are variably expressed in a variety of cancers, and many investigations have demonstrated that each type of tumor has a unique pattern of DR expression. For instance, DRD3 is only expressed in glioblastoma and is expressed at lower levels than in a healthy brain, while DRD4 is overexpressed only in acute myeloid leukemia (AML). Changes in the expression of DRs have been noted not only in cancer cells but also in cells linked to tumors. Additionally, forcing changes in the expression of DRs changes a variety of cancer cell functions, suggesting that altering the function of the receptors affects tumor biology. In order to enhance clinical response in cancer patients, DRs were suggested as prospective therapeutic targets; however, it is currently unclear how DR-targeting medications may be used in cancer treatment [8].

Rosas-Cruz et al. (2021) found that the majority of the medications under investigation have moderate or poor specificity, indicating that the biological effects seen may be caused—at least in part—by the drugs’ activity on other DRs, other GPCRs, or other cellular targets. For instance, the DRD2 antagonist pimozide, which is used as a second-line treatment for Tourette’s disorder and was proven to have anticancer properties, can also bind to alfa receptors, the serotonin receptor 5-HT7, or calmodulin. Some medications that target DRs offer features that make them attractive candidates for creating new anticancer therapeutics, notwithstanding their decreased specificity [8].

## 4. Repurposed Drugs for Oncology Using Dopamine as the Study Pathway

Given the numerous difficulties in developing novel anticancer treatments, repurposing drugs is a viable strategy for combating cancer. This approach refers to the application of a drug for a different indication than the one it was originally approved for. It has several advantages and has received increasing interest as an alternative strategy to the synthesis of new drugs. 

More than 40 years ago, the first demonstration of dopamine’s impact on tumor cells was observed when dopamine inhibited the growth of B16 melanoma cells, but the mechanisms of this inhibition remain undiscovered [14]. Since this study, the effect of dopamine on tumor cells and on cancer treatment itself has become more interesting, and thus, several studies have already shown molecular evidence of the mechanisms of dopamine activity in cancer. 

### 4.1. The Twelve Promising Targets/Drugs

**Figure 3:**
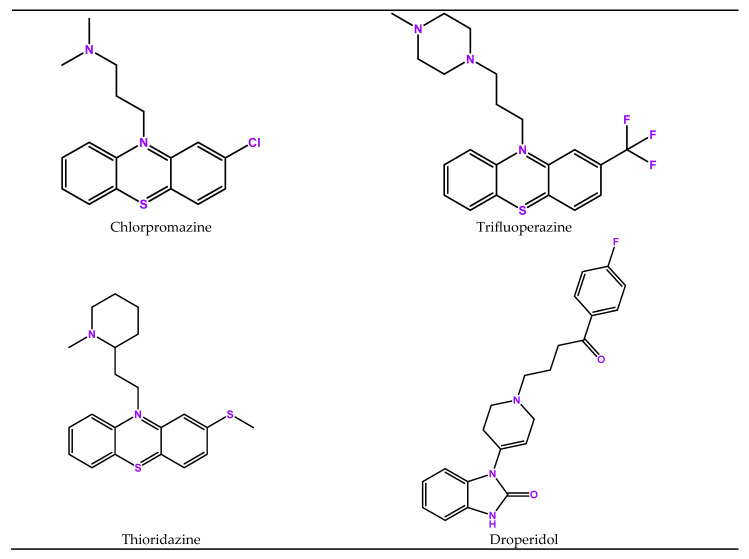

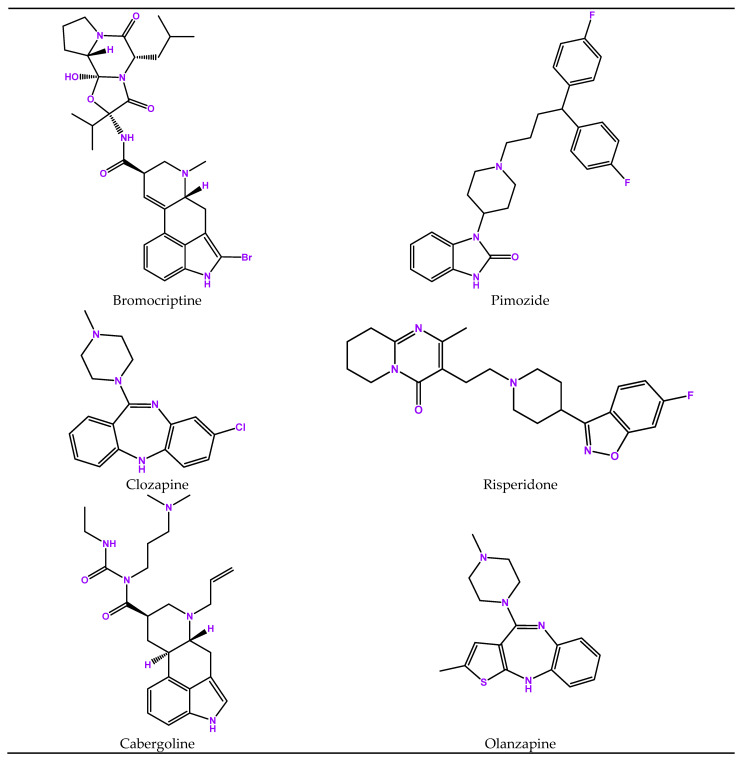

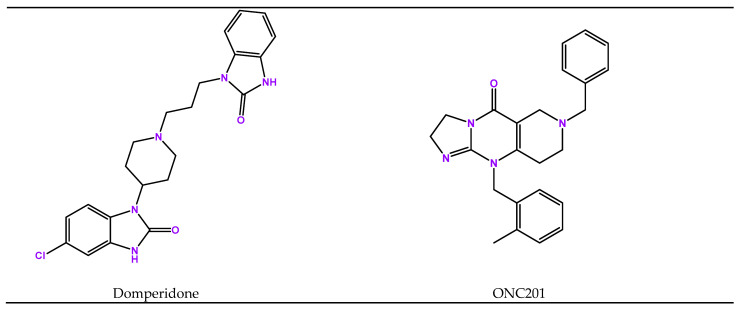
Structures of twelve drugs repurposed for oncology that use serotonin as target.

The twelve drugs that are best reused in oncology using dopamine as a target and its receptors are shown in Figure 3.

### 4.2. Chlorpromazine (Thorazine)

Chlorpromazine (CPZ) is a member of the thiazine class of heterocyclic compounds known as phenothiazines. It was the first antipsychotic to be discovered and revolutionized the treatment of psychiatric disorders (completely changed how psychiatric diseases were treated), being approved by the Food and Drug Administration (FDA) in 1957 [15]. Initially, it was believed that the serotonin receptors’ inactivation was what caused the antipsychotic effects of CPZ. Nevertheless, later research established a paradigm that is still accepted today, according to which the main cause of the antipsychotic effect of CPZ is the inhibition of the dopamine D2 receptors. In this way, the CPZ has a higher specificity for dopamine receptors and a higher affinity for dopamine D2 receptors. Later studies also showed that the CPZ inhibits dopamine’s ability to bind to receptors for dopamine D2 in the brain regions involved in controlling emotional behavior [15]. The antagonist action of CPZ reduces the symptoms of psychotic disorders such as schizophrenia, bipolar disorder, and psychotic disorders that are linked to increased dopaminergic signaling. The use of CPZ as an antipsychotic drug led to further studies on its effect on new cellular targets, and it became evident that CPZ affected several unrelated cellular targets and processes, such as the inhibition of DNA synthesis, suppression of adenosine triphosphatase (ATPase) enzyme activity, inhibition of lipase activity, and alteration of membrane permeability. Thus, all the new targets/mechanisms that were discovered for CPZ are mostly involved in either cancer development or progression, which has led to the study of this drug as a drug with anticancer activity. Accordingly, several studies were conducted on the effect of CPZ on cancer, and it was observed that this drug has an antiproliferative effect on different types of cancer (such us breast cancer [16], colorectal cancer [17], brain tumors [18], skin cancer [19], leukemia and lymphoma [20], and lung cancer [21]), which is achieved through its effect on different molecular targets and cellular pathways. Due to its known sedative effect, CPZ offers an additional advantage that helps lessen cancer patients’ feelings of anxiety, sleeplessness, and other mental health issues [15].

### 4.3. Trifluoperazine

Trifluoperazine (TFP) is an antipsychotic drug derived from phenothiazine, approved by the FDA in 1959 and considered a high-potency antipsychotic due to its dopamine receptor blocking effect [22,23,24]. TFP is used in the treatment of psychiatric disorders, in higher doses, mainly in the treatment of schizophrenia. Since low-potency drugs have a lower affinity for dopamine receptors, a higher dose is needed for the effective treatment of the symptoms of these disorders. It can also be used, but in lower doses, to treat nausea and vomiting [24]. This drug reduces the symptoms of schizophrenia, such as delusions and hallucinations, by inhibiting dopamine D1 and D2 receptors in the mesocortical and mesolimbic pathways [24]. Several pathways, including DNA damage repair, the Wnt/β-catenin pathway, the mitogen-activated protein kinase (MAPK) pathway, and retinoic acid receptor signals, were implicated in the anticancer processes of phenothiazine medications [23]. As a result, increasing research demonstrates that TFP, either by itself or in combination with other chemotherapeutic drugs, can successfully cause tumor cell death [23]. Therefore, TFP as a metastatic agent already demonstrated preclinical efficacy in triple-negative breast cancer, glioblastoma, pancreatic ductal adenocarcinoma, lung cancer, and colorectal cancer [9]. Furthermore, by causing cell cycle arrest at G0/G1, DRD2 inhibition with TFP significantly reduces the development of CRC cell lines in vitro and in vivo. TFP also targets the DR-pathway-associated components calmodulin and Forkhead Box Protein O1 (FOXO1), as well as mitochondria-mediated intrinsic apoptosis. In a mouse GBM model, TFP was most recently demonstrated to improve the effectiveness of radiation therapy by reducing the phenotypic conversion of glioma starting cells to transformed cells [9]. Thus, these studies have shown that trifluoperazine will be a promising candidate for drug repurposing in the context of cancer treatment.

### 4.4. Thioridazine

Thioridazine (TZ), an antipsychotic drug, is a dopamine receptor antagonist with a higher affinity for the dopamine D2 receptor (DRD2) was approved by the FDA in 1962 [9,25]. Thus, DRD2 could be a promising therapeutic target for cancer therapy, according to previous studies, and there are also numerous studies already demonstrating the anticancer effect of this drug and its ability to induce apoptosis in several tumor lines [25]. It was shown that TZ selectively targets cancer stem cells (CSCs) by acting to enhance CSC differentiation rather than killing these cells, contrary to how other drugs act [26]. Therefore, dopamine receptor antagonists, such as TZ, may be therapeutic targets due to the high expression of DRs in CSCs [26]. Furthermore, TZ has antiproliferative activity and apoptosis-inducing effects on various tumor cell lines and CSCs. It also makes multidrug-resistant cancer cells sensitive to cytotoxic drugs to which they have already been resistant [25]. TZ can cause apoptosis and necrosis in human uterine cervical cancer cells and limit their ability to proliferate. In cervical and endometrial cancer cells, TZ can suppress the PI3K/Akt/mTOR/p70S6K signaling pathway, leading to cell cycle arrest and apoptosis, and it was shown that leukemic cancer cells undergo apoptosis when exposed to TZ. In addition, this drug induces B16 melanoma cells to go into apoptosis and exhibit antitumor action in vivo, and it can interfere with DNA damage responses and DNA repair [25].

### 4.5. Droperidol

Droperidol, a butiferone derivative, was approved by the FDA in 1970 and is an antiemetic drug used in medicine for the control of psychosis and agitation, for vertigo, and for the treatment of benign headaches. In Europe, it is most often used to treat acute and chronic psychosis while in the US it is mainly used as antiemetics [27,28]. Although droperidol was one of the most common antiemetic medications in surgical areas for decades, the FDA increased its concerns surrounding its usage in 2001, issuing one of the most significant warnings for an FDA-approved drug. Droperidol use was linked to QT segment prolongation and/or torsades de pointes, which in some cases culminated in a fatal cardiac arrhythmia, according to the FDA [29]. However, the effect of droperidol antiemetic doses on cardiac arrhythmia remains uncertain, and the FDA’s decision is being contested [29]. We can presume that the cardiac risk of droperidol is dosage dependent, thus there is a growing trend to use the lowest effective doses of these medications, as is already done with other drugs with extremely severe side effects (Artigo 2). Droperidol is active at many different biochemical locations, particularly as a dopamine (D2) receptor antagonist [28]. With regard to the use of this drug as a repurposed cancer drug, no study has yet demonstrated its anticancer activity for the treatment of cancer, and thus, further studies are needed in this area regarding the use of droperidol.

### 4.6. Bromocriptine

Bromocriptine is a dopamine D2 receptor agonist alkaloid, which was approved by the FDA in 1978 and was widely used to treat Parkinson’s disease, galactorrhea, type II diabetes mellitus, and hyperprolactinemia [30]. By binding to dopamine receptors, this drug will suppress synthesis and thus suppress prolactin release from the anterior pituitary, reducing serum concentrations of this hormone [31]. Through the dopamine receptor, bromocriptine can also directly modify T and B cells. As a result, bromocriptine appears to be useful in the treatment of mild to severe rheumatic and auto-immune illnesses because of its inhibitory characteristics against the immune-stimulating hormone prolactin and its direct actions on the T and B ligands [31]. In some studies, it was shown that bromocriptine was able to suppress the growth of MCF-7 (breast cancer cells) in a concentration-dependent manner and was also able to inhibit the proliferation of Hepa1-6, SMMC-7721 and HCC-LM3 (hepatocellular carcinoma cells) in a concentration-dependent manner [5]. In addition to these results, it was also shown that this drug was able to inhibit the growth of xenografts from non-small cell lung cancer patients in a D2-dopamine-receptor-dependent manner and, more recently, this drug was identified as a good candidate for the treatment of acute myeloid leukemia by the FDA [9]. These results demonstrate that bromocriptine could be a reused drug in cancer treatment in the future.

### 4.7. Pimozide

Pimozide, a dopamine D2 receptor blocking agent, was approved in 1984 by the FDA and is used to treat Tourette’s syndrome, chronic psychosis, resistant phonic, and motor tics [32,33]. By inhibiting dopaminergic, serotoninergic, and other unknown CNS receptors, this drug affects CNS neurons. And because of its affinity for the HERG channel, pimozide exhibits little or no selectivity for D2 or 5-HT2A receptors. This lack of selectivity leads to several secondary changes in metabolism and central dopamine function, which have beneficial effects on resistant phonic tics and symptoms of schizophrenia and psychosis [32]. Several studies have already shown that pimozide is a potential anticancer drug, and good results have been observed in all studies using pimozide as an anticancer drug, especially regarding CSCs responsible for chemoresistance [32]. Thus, pimozide has already shown antiproliferative effects in a variety of tumor cell lines, both in vitro and in vivo, such as breast (MCF-7, T47D, ZR75-1B, MDAMB-231), lung (NSCLCA549, H460, HCC4006, e H1437), neuroblastoma, colorectal, pancreatic and hepatocellular carcinoma, melanoma, prostate, ovary, myeloproliferative neoplasms, osteosarcoma, and CNS tumors [32,33]. These antiproliferative effects may be due to several molecular targets—such as reduced phosphorylation level of STAT5, cell cycle arrest in G0/G1 phase and decreased levels of STAT3, decreased expression of Bcl-2, inhibition of MAPK pathway and Ca^2+^ channels, and suppression of ERK signaling [33,34]—that lead pimozide to be able to act effectively on tumor cells.

### 4.8. Clozapine

Clozapine, a tricyclic dibenzodiazepine, is a drug that was approved by the FDA in 1983 and is still considered the most effective antipsychotic for the treatment of therapy-resistant schizophrenia [35,36]. Despite being extremely successful, clozapine is still not widely administered due to its serious side effects and the need for frequent laboratory monitoring [37]. Clozapine has two approved uses, according to the FDA. The first is for patients with treatment-resistant schizophrenia who have previously used it, and the second is to reduce the risk of self-harm in patients with suicidal behaviors [37]. This drug is a dopaminergic receptor antagonist, with a high affinity for D4 and a low affinity for D2 receptors. It also binds to multiple serotonin receptors with a high affinity [37], which points to these receptors as being responsible for the efficacy of this drug as an antipsychotic. Despite all of the above, and the fact that this drug is known to affect many neurotransmitters in the brain, its exact mechanism of action remains to be discovered [37]. In one study, it was shown that clozapine was able to reduce the number of acute myeloid leukemia (AML) cells after treatment with the drug, thus demonstrating anticancer activity in this tumor cell line [38].

### 4.9. Risperidone

Risperidone, a benzisoxazole derivative, was the first second-generation antipsychotic to be developed and is a potent antagonist of the dopamine D2 and serotonin 5-HT2 receptors found in the brain, with the 5-HT2A antagonism being significantly stronger than the D2 antagonism [39,40,41]. This drug was approved by the FDA in 1993 and is indicated for the acute and maintenance treatment of schizophrenia and other related psychotic disorders, including acute bipolar mania [39]. The therapeutic activity of a dose of risperidone is therefore represented by the combined effects of each substance, since the primary metabolite of each substance, 9-hydroxy-risperidone, has a similar receptor binding activity [39]. There is a large body of evidence proving efficacy in treating positive and negative symptoms, as well as a better EPS tolerability profile than standard neuroleptics, especially at lower doses [40]. Risperidone has already demonstrated cytotoxic properties on proliferating gastric cancer cells and has also shown neurotoxic and neuroprotective activity in neuroblastoma cells (SK-N-SH cell) [42,43]. In colorectal cancer cells, risperidone also showed the ability to decrease the viability of these cells [44]. These findings thus demonstrate anticancer characteristics of risperidone in different tumor cell lines.

### 4.10. Cabergoline

Cabergoline (CAB) is a synthetic ergoline derivative with dopamine agonist activity that was approved by the FDA in 1996 [45,46]. This drug has a high selectivity and affinity for the D2 dopamine receptor and also has a potent, selective, and long-lasting inhibitory activity on the secretion of prolactin, a hormone that acts on the neurotransmitter receptors present on the pituitary lactotrophs [45,46]. It is marketed for the treatment of PD and hyperprolactinemia and has unique pharmacokinetic and pharmacodynamic properties that make it distinguishable from other dopamine agonists [46]. It was demonstrated that CAB, an ergot derivative, can normalize serum prolactin levels in patients with dysregulated prolactin levels by having a potent and long-lasting effect of reducing prolactin secretion both in vitro and in vivo in various animal models [45]. Thus, in addition to neurological diseases, BAC can be used as a first-line treatment in patients with micro- and macroprolactinomas, that is, in patients with benign pituitary tumors that are prolactin-secreting—and also in other hyperprolactinemic disorders [46]—because it has a high antitumor effect on these tumors and optimal patient tolerance [45]. Other studies have demonstrated that CAB can be used to treat various pituitary adenomas, including those that secrete growth hormone and ACTH [45]. As shown in previous studies, the functional ratio between ERK1/2 and PI3K pathways is altered in prolactinoma cells by cabergoline-induced DRD2 agonization, which consequently will inhibit PI3K/AKT/mTOR-mediated proliferation and promote ERK/S6K-mediated differentiation [9]. Given the significance of DRD2 and DA agonists in regulating PRL secretion and milk production, it is also crucial to comprehend the significance of this pathway in breast cancer [9]. The fact that many antipsychotics enhance blood levels of PRL via antagonizing lactotrope inhibitory D2Rs may help to explain why there is a higher risk of breast cancer in female schizophrenia patients. Thus, there are already phase I, II, and III studies that have tested the role of cabergoline in metastatic breast cancer and the efficacy of cabergoline in individuals with non-functioning pituitary adenomas [47,48].

### 4.11. Olanzapine

Olanzapine, approved by the FDA in 1996, is a thienobenzodiazepine derivative with a structure close to that of clozapine, and it is classified as an atypical antipsychotic [49,50]. This drug has the ability to bind to different types of dopaminergic (D1, D2, D3, D4, and D5) and serotoninergic (5HT2A/2C, 5HT3, 5HT6, and 5HT7) receptors, and it has a higher affinity for 5HT2 receptors compared to dopamine receptors [50,51]. It is an antipsychotic, antimanic, and stabilizer used for the treatment of manic episodes, schizophrenia, and bipolar disorder [50]. It has a variety of advantages in the treatment of patients with advanced cancer since it inhibits the activity of certain G protein-coupled receptors as well as other receptors [49]. In this way, olanzapine was shown in earlier studies to be a safe and effective treatment for symptoms connected to cancer, such as chemotherapy-induced nausea and vomiting, cancer pain, anorexia, and cachexia [51]. In previous studies, it was demonstrated that olanzapine sensitizes pancreatic CSCs to chemotherapeutic agents in vitro [8] and that the combination of olanzapine plus temozolomide, an alkylating agent, inhibits the growth of glioma cell lines more effectively than either alone [52].

### 4.12. Domperidone

Domperidone is a benzimidazole dopamine D2 receptor antagonist, which has never been approved by the FDA because it has shown adverse effects, such as risk of cardiac arrhythmias in cancer patients treated with high doses of this drug, but it has been available in several countries for several years [53,54]. This drug is used to treat nausea and vomiting [55]. Although it acts in regions of the CNS that do not have a blood–brain barrier, such as those that control prolactin production, it is difficult for this drug to penetrate the blood–brain barrier. In the liver and intestinal wall, domperidone is metabolized primarily and largely by cytochrome P450 3A4 (CYP3A4) [53]. Dopamine inhibits tumor development and lung metastasis in vivo in a DRD2-dependent manner since the effects are reversed when dopamine and the DRD2 antagonist domperidone are given at the same time [8]. In a previous study, it was demonstrated that domperidone can significantly abrogate DA-mediated invasion inhibition in Hepa1-6 cells, which may highlight a role for this drug in liver carcinoma cell proliferation and invasion [56].

### 4.13. ONC201

ONC201 is a chemical compound that was recently discovered as a first-in-class TNF-related apoptosis (TRAIL)-inducing compound (TIC10) [57,58]. ONC201 appears to be an allosteric agonist of the caseinolytic protease P (ClpP) and a selective competitive and non-competitive antagonist of the dopamine D2 receptor (DRD2), a GPCR that is over-expressed in a variety of malignancies. Therefore, DRD2 antagonists that are stronger than ONC201 are not more potent anticancer drugs for dopamine receptors compared to this drug [58]. These effects cause antiproliferative and/or pro-apoptotic phenotypes in a cellular context-dependent manner. In all preclinical models, neither DRD2 nor ClpP alone can predict the ONC201 response [58]. This substance promotes the integrated stress response (ISR), OXPHOS, Akt/ERK activation, degradation of c-myc, and induction of the DR5/TRAIL receptor in tumor cells. In tumor cells with high levels of DRD2 and/or ClpP expression, or a dependence on other components of the mechanism of action (e.g., c-myc or OXPHOS), these effects cause antiproliferative and/or pro-apoptotic phenotypes in a cellular context-dependent manner. In all preclinical models, neither DRD2 nor ClpP by themselves can forecast how ONC201 will react [58]. Inhibition of cancer stem cell self-renewal, activation of natural killer cells, and adverse effects caused by fibroblasts in the tumor microenvironment have all been linked to treatment with ONC201. The preclinical activity of ONC201 was shown in a variety of advanced solid tumors and hematologic neoplasms as a single agent and in synergistic combinations with radiation, chemotherapy, targeted therapy, and immunotherapy. This is consistent with the broad relevance of ONC201′s mechanism of action in many types of cancer [58]. The clinical use of this drug in the treatment of different types of cancer continues to be studied and evaluated in various clinical trials to achieve its proof. The anticancer properties of ONC201 have been demonstrated in a variety of preclinical tumor models, including breast cancer cell lines, CRC cells, glioblastoma cell lines and patient-derived glioblastoma cells, primary human lung cancer cells, endometrial cancer cells, prostate cancer tumor in vitro, hepatocellular carcinoma cells, and hematologic malignant tumors (such as acute lymphoblastic leukemia, acute myeloid leukemia, Burkitt’s lymphoma, Hodgkin’s lymphoma, and multiple myeloma cell lines) [9,58]. 

## 5. Conclusions

These data serve as a reminder that in order to appropriately analyze the efficacy and molecular mechanism of action within each kind of cancer, the tissue-specific effects of DRs and their pharmacological ligands must be explained in detail. The therapeutic application of antipsychotics for the treatment of cancer requires more research and development even if it was acknowledged that certain types of them have potential anticancer action.

This type of investigation is dynamic, and several approaches are needed to identify possible candidates to be reused. Research in this area is constantly evolving, and new discoveries may provide more detailed information about the role of dopamine and other neurotransmitters in the repurposing of drugs to treat cancer. As an example, we can highlight the importance of investigating drug metabolism and combinations as areas to be explored and thus ensure greater success in the reuse of drugs for oncology.

## Data Availability

Not applicable.
